# Utility of the Simulated Outcomes Following Carotid Artery Laceration Video Data Set for Machine Learning Applications

**DOI:** 10.1001/jamanetworkopen.2022.3177

**Published:** 2022-03-21

**Authors:** Guillaume Kugener, Dhiraj J. Pangal, Tyler Cardinal, Casey Collet, Elizabeth Lechtholz-Zey, Sasha Lasky, Shivani Sundaram, Nicholas Markarian, Yichao Zhu, Arman Roshannai, Aditya Sinha, X. Y. Han, Vardan Papyan, Andrew Hung, Animashree Anandkumar, Bozena Wrobel, Gabriel Zada, Daniel A. Donoho

**Affiliations:** 1 Department of Neurosurgery, Keck School of Medicine of the University of Southern California, Los Angeles; 2 Department of Computer Science, Viterbi School of Engineering, University of Southern California, Los Angeles; 3 Department of Operations Research and Information Engineering, Cornell University, Ithaca, New York; 4 Department of Mathematics, University of Toronto, Toronto, Ontario, Canada; 5 Center for Robotic Simulation and Education, USC Institute of Urology, Keck School of Medicine of the University of Southern California, Los Angeles; 6 Department of Computer Science and Mathematics, California Institute of Technology, Pasadena; 7 Department of Otolaryngology, Keck School of Medicine of the University of Southern California, Los Angeles; 8 Division of Neurosurgery, Center for Neuroscience, Children’s National Hospital, Washington, DC

## Abstract

**Importance:**

Surgical data scientists lack video data sets that depict adverse events, which may affect model generalizability and introduce bias. Hemorrhage may be particularly challenging for computer vision–based models because blood obscures the scene.

**Objective:**

To assess the utility of the Simulated Outcomes Following Carotid Artery Laceration (SOCAL)—a publicly available surgical video data set of hemorrhage complication management with instrument annotations and task outcomes—to provide benchmarks for surgical data science techniques, including computer vision instrument detection, instrument use metrics and outcome associations, and validation of a SOCAL-trained neural network using real operative video.

**Design, Setting, and Participants:**

For this quailty improvement study, a total of 75 surgeons with 1 to 30 years’ experience (mean, 7 years) were filmed from January 1, 2017, to December 31, 2020, managing catastrophic surgical hemorrhage in a high-fidelity cadaveric training exercise at nationwide training courses. Videos were annotated from January 1 to June 30, 2021.

**Interventions:**

Surgeons received expert coaching between 2 trials.

**Main Outcomes and Measures:**

Hemostasis within 5 minutes (task success, dichotomous), time to hemostasis (in seconds), and blood loss (in milliliters) were recorded. Deep neural networks (DNNs) were trained to detect surgical instruments in view. Model performance was measured using mean average precision (mAP), sensitivity, and positive predictive value.

**Results:**

SOCAL contains 31 443 frames with 65 071 surgical instrument annotations from 147 trials with associated surgeon demographic characteristics, time to hemostasis, and recorded blood loss for each trial. Computer vision–based instrument detection methods using DNNs trained on SOCAL achieved a mAP of 0.67 overall and 0.91 for the most common surgical instrument (suction). Hemorrhage control challenges standard object detectors: detection of some surgical instruments remained poor (mAP, 0.25). On real intraoperative video, the model achieved a sensitivity of 0.77 and a positive predictive value of 0.96. Instrument use metrics derived from the SOCAL video were significantly associated with performance (blood loss).

**Conclusions and Relevance:**

Hemorrhage control is a high-stakes adverse event that poses unique challenges for video analysis, but no data sets of hemorrhage control exist. The use of SOCAL, the first data set to depict hemorrhage control, allows the benchmarking of data science applications, including object detection, performance metric development, and identification of metrics associated with outcomes. In the future, SOCAL may be used to build and validate surgical data science models.

## Key Points

**Question:**

What is the utility of a data set that contains videos of surgeons managing hemorrhage?

**Findings:**

This quality improvement study of the Simulated Outcomes Following Carotid Artery Laceration (SOCAL), a public data set of surgeons managing catastrophic surgical hemorrhage in a cadaveric training exercise included 65 071 instrument annotations with recorded outcomes. Computer vision–based instrument detection achieved a mean average precision of 0.67 on SOCAL and a sensitivity of 0.77 and a positive predictive value of 0.96 at detecting surgical instruments from real intraoperative video.

**Meaning:**

A corpus of videos of surgeons managing catastrophic hemorrhage is a novel, valuable resource for surgical data science.

## Teaser

This quality improvement study evaluates the utility of a publicly available surgical video data set of hemorrhage complication management with instrument annotations and task outcomes.

## Introduction

Advances in machine learning and computer vision (CV) facilitate the discovery of unknown patterns in large bodies of visual data.^1,2,3,4,5^ Applying these methods to analyze surgical video from the operating room may improve patient care by detecting complications, assisting trainee development, and assessing performance.^6,7,8,9,10^ Algorithmic methods thrive when presented with comprehensive data sets. Surgical video data sets, however, are traditionally collected to answer a single question by a single group of investigators, depicting a particular surgical scenario (eg, one data set for object detection in neurosurgery and a different data set for phase detection in general surgery).^11,12,13,14,15,16,17,18,19,20,21,22,23^ This patchwork approach risks fostering data set bias, a condition that occurs when model training data are not representative of the true data landscape (ie, all surgical scenarios).^24^

We cataloged surgical video data sets into a single corpus (eTable 1 in the Supplement) and detected a clinically relevant data set bias: the absence of surgical adverse events. Because of institutional and medicolegal sensitivities, no video data sets of surgeons encountering complications, particularly bleeding events, are available, which creates a dangerous lacuna for CV algorithms. Investigators cannot develop tools to assess complication management and cannot evaluate the performance of existing tools during scenarios that compromise visualization, such as hemorrhage.

To address this gap, we recorded videos from a multiyear, nationwide educational curriculum in which surgeons of all experience levels encountered catastrophic hemorrhage in a high-fidelity, validated cadaveric model of internal carotid artery (ICA) laceration. Laceration of the ICA is a life-threatening risk inherent in anterior cranial base surgery (eg, endoscopic sinus surgery and pituitary tumor surgery) that can result in stroke, neurologic injury, or death.^25,26^ Because ICA laceration occurs in less than 0.2% of cases,^25,27^ most surgical trainees never experience this event during formal surgical training. Video of these scenarios often contains bleeding that can obscure the entire surgical scene. These cadaveric simulations allowed surgeons to hone their skills and receive feedback in a highly realistic, structured environment without risking patient harm.^28,29,30^

We used these videos to develop the Simulated Outcomes Following Carotid Artery Laceration (SOCAL) data set. SOCAL’s size, high level of realism, varied anatomy and operative views, and validated performance outcomes^30,31,32,33^ make it a unique resource. These otherwise unobtainable videos of hemorrhage complication management can support surgical data science tasks and be used to develop machine learning models. This article publicly releases the SOCAL data set^34^ and provides several illustrative cases central to surgical data science using this data set: deep learning performance benchmarking, metric development, and intraoperative video instrument detection.

## Methods

Surgeon verbal consent for video recording was obtained, and the cadaveric experiment was approved by the institutional review board of the University of Southern California. For the patient case discussed, consent was waived by the institutional review board given the study's retrospective nature and the nonidentifiable nature of the intraoperative video. This quality improvement study followed the Standards for Quality Improvement Reporting Excellence (SQUIRE) reporting guideline.

### Data Set Development

**Figure 1.**
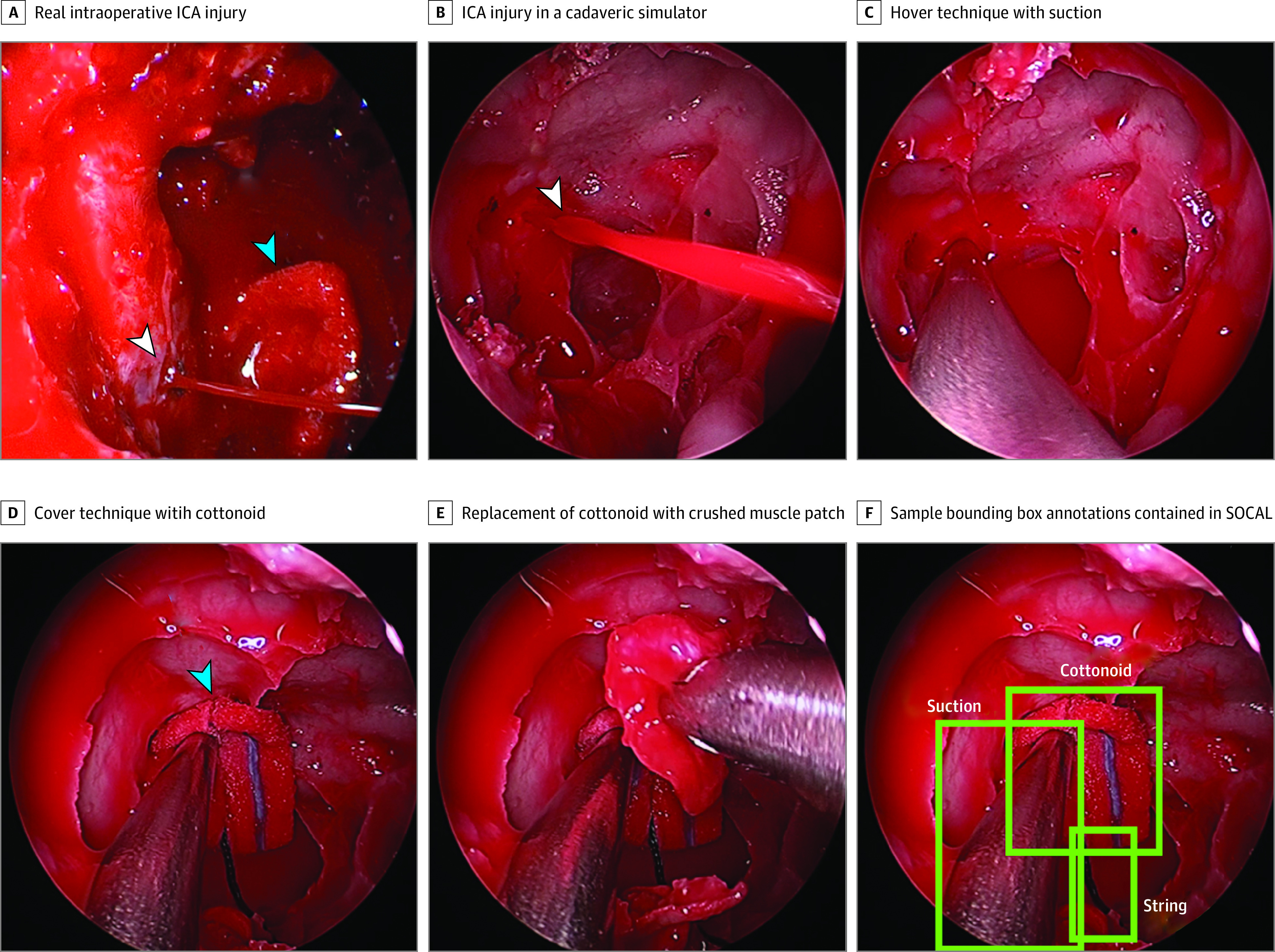
Endoscopic Images of Internal Carotid Artery (ICA) Injury Management An actual ICA and steps to achieve hemostasis in the cadaver model. Surgical instruments were hand annotated in each video frame with bounding boxes using the annotation tool Vott. White arrows indicate injury; blue arrows, instrument.

We collected videos of surgeons participating in nationwide training courses for ICA laceration management from January 1, 2017, to December 31, 2020. These courses were held at the North American Skull Base Society, Keck School of Medicine of the University of Southern California, the Emory University School of Medicine, and the Combined ENT & Neurosurgery Advanced Resident Sinus and Skull Base Dissection course in Park City, Utah (sponsored by Stryker Corp). Surgeon characteristics and task outcomes were recorded. Details of the methods,^35^ validation,^32^ costs,^31^ and results^30^ of the training course have previously been published. In brief, surgeons attempted to manage an ICA laceration in a perfused (bleeding) human cadaveric head during a standardized exercise: the first attempt was performed before coaching and the second attempt after personalized coaching instruction was delivered. A real ICA example is shown in Figure 1A, and the steps to achieve hemostasis in the cadaver model are depicted in Figure 1B-E and described in the eMethods in the Supplement. These experiments have been validated as having exceptionally high realism and transferability to the operating room.^30,31,32,33,35^

The Karl Storz Video Neuro-Endoscope records intraoperative video during each of these trials. The SOCAL data set consists of 147 annotated videos from 7 different cohorts of surgeons; trial duration varies from 46 seconds to 5 minutes. We developed the data set following previously published methods.^36^ Surgical instruments were hand annotated in each video frame with bounding boxes using the annotation tool Vott (Figure 1F).^37^

### Object Detection Algorithm and Evaluation

Our data set was used to train 2 object detection models: RetinaNet and YOLOv3.^38,39^ Both RetinaNet and YOLOv3 are convolutional neural network (CNN)–based deep neural networks (DNNs) developed to detect spatial patterns within images. RetinaNet is composed of a backbone network that computes a feature map over an input image using CNNs and 2 task-specific subnetworks that use CNNs to classify objects detected in the backbone network and perform bounding box regression. YOLOv3 is a single-stage object detection method that uses CNNs to predict bounding boxes and assign probabilities to class types from the entire image. These architectures achieved competitive results on ImageNet,^38,39^ which is a standardized data set used to benchmark and compare object detection methods.^40^ An intersection over union greater than 0.5 (ie, where >50% of the area of the detection lies within the area of the ground truth annotation) was considered a successful detection. Further details on model implementation are in the eMethods in the Supplement.

### Instrument Use Pattern Features

We generated rudimentary instrument use variables from ground truth annotations of surgical instruments. Features of interest were selected based on known ICA injury surgical progression and clinical expertise. Instrument use patterns for each key instrument in the procedure (suction, grasper, cottonoid, string, and muscle) were generated by summing the number of frames with the instrument in view and dividing that value by the total number of frames for that trial.

### Intraoperative Video Analysis

Three and a half minutes (210 seconds) of video was taken from the case of an uncomplicated endoscopic, endonasal tumor resection of a sellar craniopharyngioma (not published within SOCAL) to validate automatic tool detection in a real intraoperative setting. We parsed the video into individual frames at 0.5 frames per second (total of 105 frames). We used the RetinaNet model trained on SOCAL and validated it by predicting the location of instruments in each frame of the operative case video. The model output bounding boxes around detected instruments without specifying tool type (ie, instruments were labeled as *tool* instead of *grasper* or *suction*). A member of the research team performed ground truth annotations. Videos were annotated from January 1 to June 30, 2021.

### Statistical Analysis

Object detection model performance on SOCAL was evaluated using mean average precision (mAP) (average area under the precision-recall curve), a standard metric used to evaluate object detection networks. Object detection model performance on real intraoperative video was evaluated using sensitivity and positive predictive value for instrument detections. For performance metric associations with outcomes, we fit individual linear regression models to predict blood loss from each instrument use pattern while accounting for the time to hemostasis for that trial as a covariate and reported the effect size, variance explained, and associated *P* value of each fitted model. When analyzing instrument usage patterns, we consider a 2-sided *P* < .05 to be statistically significant.

## Results

### Data Set Description

SOCAL contains 147 videos of 75 individual surgeons (neurosurgeons and otorhinolaryngologists with 1 to 30 years’ experience [mean, 7 years]) managing a simulated life-threatening hemorrhage in a cadaveric model before and after expert coaching during a 4-year period (7 cohorts) (eTable 2 and eMethods in the Supplement).^30^ Surgeon experience ranged from junior trainees to experts (48 trainees, 25 attending physicians, and 2 not reported). Almost half of the surgeons failed to control hemorrhage on the first attempt, resulting in simulated patient mortality. Paired videos with outcome data are available for 71 surgeons (4 unpaired trials; 1 surgeon with a third trial). Thirty-one participants failed in trial 1 and succeeded in trial 2 after expert instruction. Blood loss decreased by 29% (154 mL; 95% CI, 65-243 mL) after expert instruction. Surgeon participant outcomes are given in eTable 2 in the Supplement.

A total of 31 443 frames of video were annotated with instrument-level bounding boxes across 5 surgical instruments (Table 1) for a total of 65 071 unique instrument instances. Each frame had 0 to 5 surgical instruments. An image from the SOCAL data set compared with an actual intraoperative ICA injury is shown in Figure 1, illustrating the high-fidelity nature of the simulator. A description of surgical instrument findings within the data set is given in Table 1.

**Table 1.** Simulated Outcomes Following Carotid Artery Laceration Video Data Set Instrument Instances, Training, Validation, and Test Sets

Variable	All videos	Training	Validation	Test
No. of trials	147	124	9	14
No. of frames	31 443	27 223	2292	1928
No. of instruments				
Suction	22 356	19 106	1862	1388
Grasper	15 943	13 576	1084	1283
String	11 917	9944	1214	759
Cottonoid	10 005	8491	610	904
Muscle	4560	3681	223	656
Tool	76	76	0	0
Drill	210	159	0	51
Scalpel	4	4	0	0

We followed the NeurIPS 2021 Code and Data Submission Guidelines for data set publishing to ensure accessibility.^41^ In following these guidelines, we placed the Digital Object Identifier (DOI) of the public SOCAL data set in a repository that ensures long-term preservation, makes the data set publicly available for download,^34^ and licenses the data set under a Creative Commons Attribution 4.0 International Public License. To illustrate the utility of SOCAL as a test bed for surgical data science techniques, we outline 3 use cases: benchmarking deep learning object detection, developing performance metrics from intraoperative video, and validating instrument detection using real intraoperative video.

### Benchmarks for Object Detection Using SOCAL

**Figure 2.**
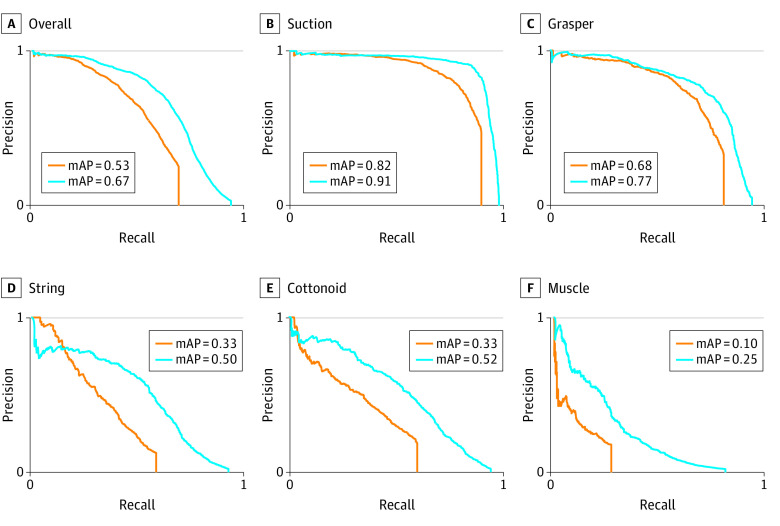
Deep Learning Instrument Detection Model Performance Orange lines represent the YOLOv3 deep neural network; blue lines represent the RetinaNet deep neural network. mAP indicates mean average precision.

We used 2 off-the-shelf object detection DNNs to demonstrate the feasibility of instrument detection using SOCAL. The overall mAPs were 0.67 for RetinaNet and 0.53 for YOLOv3. We then evaluated the mAP for each object in our data set; the results are visualized in Figure 2. The models predict suction (mAPs, 0.91 for RetinaNet and 0.82 for YOLOv3) and grasper (mAPs, 0.77 for RetinaNet and 0.68 for YOLOv3) with high accuracy. In contrast, the mAPs for cottonoid (0.52 for RetinaNet and 0.33 for YOLOv3) and string (0.5 for RetinaNet and 0.33 for YOLOv3) are lower, and the mAPs for muscle are poor (0.25 for RetinaNet and 0.1 for YOLOv3). Training, validation, and test splits and outcomes are given in Table 2.

**Table 2.** Preliminary Instrument Use Patterns and Corresponding Variance of Blood Loss

Instrument use pattern feature	Effect size	Variance of blood loss	*P* value
Time to hemostasis	2.9	49.84	.001
Frames with			
Grasper	–679.5	6.24	.001
Cottonoid	–684.6	6.18	.001
Muscle	–466.2	1.99	.02
Suction	–403.5	1.71	.03
String	–259.3	1.37	.05

### Benchmarks for Instrument Use Patterns Using SOCAL

As a feasibility demonstration of objectively describing instrument movement, instrument use patterns were calculated from on-screen bounding box location data for the 5 critical instruments used in this simulation (suction, grasper, cottonoid, string, and muscle). We found that the following features accounted for a significant amount of the variance in blood lost: the proportion of frames with grasper in use, the proportion of frames with cottonoid in use, the proportion of frames with muscle in use, and the proportion of frames with suction in use (Table 2). Time to hemostasis alone accounts for 49.84% (effect size, 2.9; *P* < .001) of the variance in blood lost.

### Instrument Detection in a Real Intraoperative Video

**Figure 3.**
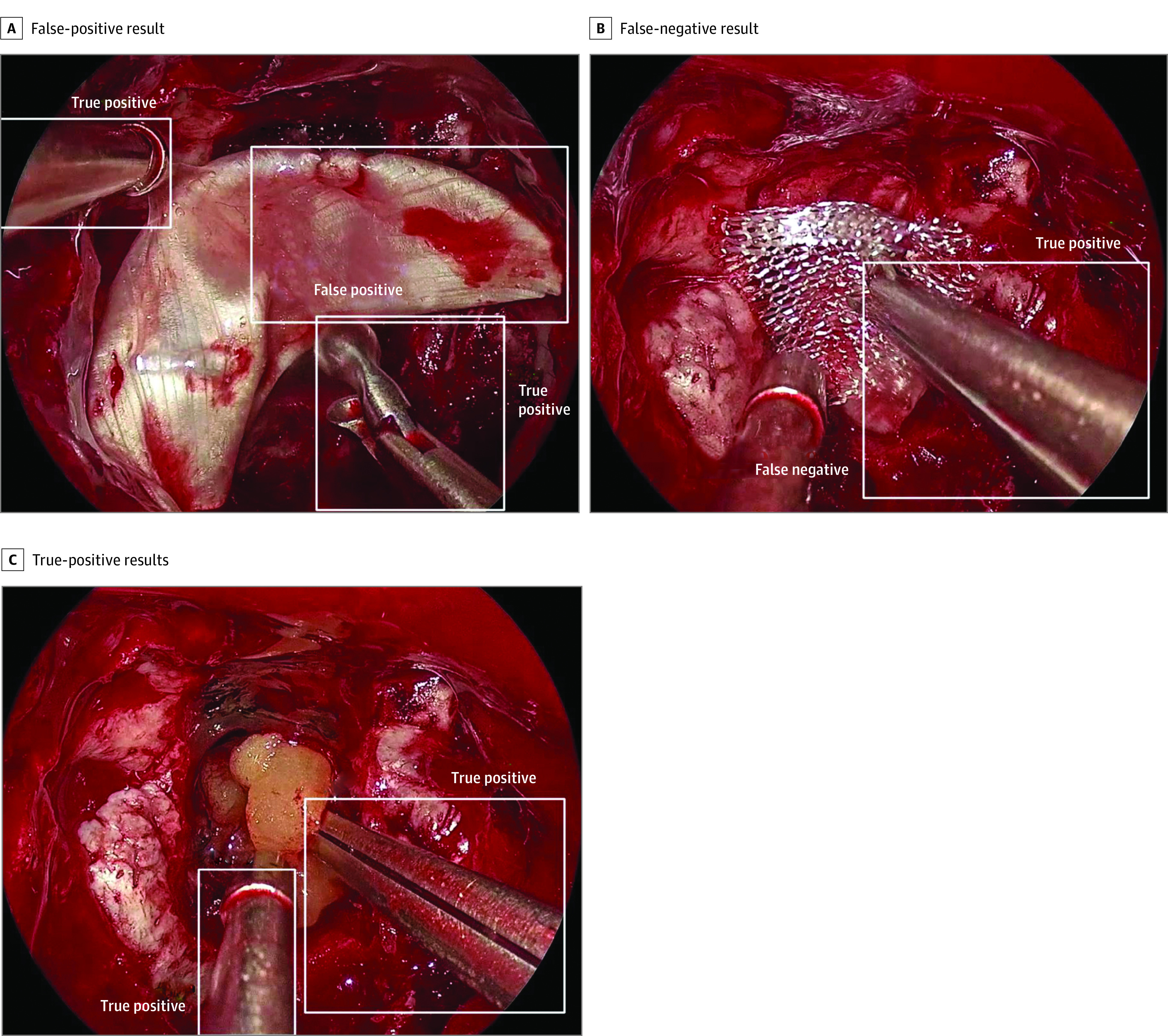
Instrument Detection Results A total of 138 instruments were identified (true-positive results), 6 noninstruments were identified (false-positive results), and 41 instruments were missed (false-negative results).

As a proof-of-concept for the SOCAL database to serve as training data for video from real patient cases, a 3-minute intraoperative video of endoscopic resection of a tumor of the anterior cranial base was analyzed. One hundred seventy-nine unique instances of surgical instruments were annotated as ground truth data. The RetinaNet model that was trained using the SOCAL data set accurately detected 138 instruments (sensitivity, 0.77). There were 41 instances of missed instruments. There were 6 instances of double annotations or mislabeling of noninstruments as instruments (positive predictive value, 0.96). Examples of true-positive, false-positive, and false-negative results are shown in Figure 3. Fascia (Figure 3A) and absorbable hemostatic agent (Figure 3B) were not counted as surgical instruments.

## Discussion

In this quality improvement study, we introduce SOCAL, a novel video data set of surgeons managing catastrophic hemorrhage. No public video data sets that depict surgical adverse events are available, and it is unlikely that a similar data set of intraoperative hemorrhage on actual patients (instead of cadavers) could ever be created. We illustrate the utility of SOCAL as a valuable resource for surgical data science by describing 3 use cases that showcase the importance of novel test bed data sets, the importance of studying surgical adverse events, and the need for additional public data sets of surgical video.

### Use of Video Data Sets as a Test Bed for Surgical Data Science Methods

SOCAL offers a test bed for methods that analyze intraoperative video and can stress test CV models because it depicts relevant and challenging conditions not present in other data sets (eg, object obscuration by bleeding). After training and testing 2 detectors (RetinaNet and YOLOv3) on the SOCAL data set, we found clinically relevant decrements in the performance of object detection in the setting of massive hemorrhage. Rigid metal objects (eg, suction and grasper) were more easily detected than malleable and discolored objects (eg, cottonoid and string). These decrements were not seen in frames without bleeding. We obtained comparative evaluations of the 2 networks and were able to identify the best-performing DNN within this context. The overall performance of these models using SOCAL can now be compared with previous results of surgical detection in lower-fidelity or more constrained surgical tasks.^15,21,42,43^ Validation using the SOCAL video may allow for future stepwise improvements in the validity of surgical object detection.

SOCAL can be used to perform core machine learning tasks, such as instrument detection and instrument movement description.^7^ We show that even rudimentary instrument movement characteristics (eg, seconds that the suction instrument is visible) are associated with operative performance. These rudimentary benchmarks will be improved by more sophisticated models that quantify surgical movement to describe surgical signatures.^7,13^

We validated a SOCAL-trained DNN using real case video, demonstrating a new pathway for augmenting small surgical data sets. This transfer learning approach uses larger surgical data sets to train and test a model and then validates findings on a smaller set of intraoperative video use cases. Laboratory or simulation data sets may be promising for model training and testing because we can replicate adverse events in high-fidelity simulation without waiting for patients to be harmed, and these videos may be easier to produce at scale. Then, once trained on simulator-based data, models can be validated on a markedly smaller number of operative video frames, solving for institutional and medicolegal sensitivities.

### Instrument Annotations and Outcome Labels in Surgical Video Data Sets

SOCAL includes data on surgeon characteristics and performance. Throughout surgical data science, researchers are interested in exploring the associations among surgeon characteristics, tool movement, task outcome, and, eventually, patient outcome, complication occurrence, and technical competency.^44^ Surgical video is the primary data source for this information for most operations aside from a few robotic-assisted procedures.^45,46,47^ Despite its importance, no publicly available data sets exist of surgical video labeled with clinically meaningful outcomes.

Using SOCAL, we can quantify the association between instrument use patterns and task outcomes and provide this feedback to surgeons. The criterion standard for feedback to surgeons is expert review, but the scarcity of experts limits scalability.^1,48,49,50^ The generation of summaries of surgical actions using video-based instrument use patterns combined with other objectively validated rating scales^51^ may provide surgeons with meaningful feedback. SOCAL depicts a wide heterogeneity of surgeon skill levels from junior trainees to world experts and contains paired records of surgeons before and after expert coaching. Investigators can use SOCAL to explore the association between surgeon experience and outcomes and between surgeon coaching and changes in instrument movement. SOCAL illustrates the need for future data sets to contain these labels.

### Studying Surgical Adverse Events 

SOCAL is the only instrument-annotated surgical video data set that depicts catastrophic hemorrhage, a surgical adverse event. Hemorrhage control tasks are valuable to study because they are life threatening and occur in all surgical fields.^52,53,54^ These tasks are well suited for CV methods because their outcomes can be labeled as success or failure and blood loss can be quantified.^55,56^ Hemorrhage also introduces a degree of realism and camera obscuration that is not captured in benchtop simulators or routine procedures.^11,22^ Finally, a direct association is seen between surgical skill and ability to achieve hemostasis, which is often exacerbated during high-stakes complication management when surgeons may potentially deviate from standard techniques because of stress, inexperience, or lack of specific skills.^30,35^ Using SOCAL, we saw that neither surgeon confidence nor experience was strongly associated with task success or blood loss in this hemorrhage control task,^30^ whereas surgeon instrument use characteristics were strongly associated with task success and blood loss.

Although the specific hemorrhage conditions observed in SOCAL are rare, hemorrhage control is crucial for all surgical procedures. Accordingly, SOCAL-validated detectors may have improved performance in the setting of hemorrhage or extensive blood product deposition as can be seen in cardiac, vascular, and some neurosurgical scenarios.

### Need for Publicly Available Data Sets 

The current landscape of surgical video analysis consists of individual surgeon-research teams that developed databases for intraoperative video analysis with limited overlap in size, aims, types of videos recorded, annotation styles, and overall functionality (eTable 1 in the Supplement). The development of intraoperative video databases faces many challenges. There is often a gap between surgeons who hold intraoperative video and engineers who can use these data. Converting raw video into an annotated, usable data set is tedious and expensive. Even if stripped of protected health information, intraoperative video may be prone to institutional or individual resistance to sharing. We hope to overcome the limitations of other data sets by making the video, annotations, outcomes, and code for development publicly available as scaffolds for other groups.

We followed the NeurIPS 2021 Code and Data Submission Guidelines such that our data set adheres to best practices and ensures accessibility and reproducibility of results.^41^ We encourage further exploration by other groups and hope the release of this data set spurs the development of other intraoperative video data sets.

### Limitations

This study has several limitations. The analysis presented serves to illustrate the utility of the SOCAL data set and leaves much room for further development of object detectors, performance metric evaluation, and outcomes associations. Many novel DNN architectures have been described in the literature but are not validated in the setting of bleeding. SOCAL could serve as a test bed for these models in future work (eg, more advanced object detection systems that use temporal sequences [long short-term memory and 3-dimensional CNN^5,57^]). Hyperparameters, such as intersection over union threshold and frame rate for detection, were not thoroughly explored in this pilot analysis and require further experimentation. SOCAL only contains data from endoscopic endonasal neurosurgery; other surgical fields may have unique instruments and camera characteristics that could challenge SOCAL-trained networks. Further description and clinical characterization of the detected features have yet to be performed.

## Conclusions

The lack of videos of adverse events creates a data set bias that hampers surgical data science. SOCAL, the first publicly accessible video data set of surgeons managing catastrophic hemorrhage, was developed to meet this need. SOCAL includes instrument annotations, surgeon experience data, and task outcomes. SOCAL serves as a unique test bed for surgical data science techniques, such as object detection, performance metric derivation, and outcome associations using deep learning models.

## Notes

Supplement. **eMethods.** Supplemental Methods **eTable 1.** Publicly Available Tool-Annotated Surgical Video Data Sets Across Surgical Specialties **eTable 2.** Baseline Demographic and Outcome Characteristics of Surgeon-Trials Contained in the SOCAL Video Database
