# Supplementary material for: Utility of the Simulated Outcomes Following Carotid Artery Laceration Video Data Set for Machine Learning Applications
Source: JAMA Netw Open. 2022 Mar 21;5(3):e223177. doi: 10.1001/jamanetworkopen.2022.3177 (PMC8938712; doi:10.1001/jamanetworkopen.2022.3177)
Supplement: Supplement. — eMethods. Supplemental Methods eTable 1. Publicly Available Tool-Annotated Surgical Video Data Sets Across Surgical Specialties eTable 2. Baseline Demographic and Outcome Characteristics of Surgeon-Trials Contained in the SOCAL Video Database [file jamanetwopen-e223177-s001.pdf]

Supplementary Online Content

Kugener G, Pangal DJ, Cardinal T, et al. Utility of the simulated outcomes following carotid artery laceration video data set for machine learning applications. *JAMA Netw Open*. 2022;5(3):e223177. doi:10.1001/jamanetworkopen.2022.3177

eMethods. Supplemental Methods

eTable 1. Publicly Available Tool-Annotated Surgical Video Data Sets Across Surgical Specialties

eTable 2. Baseline Demographic and Outcome Characteristics of Surgeon-Trials Contained in the

SOCAL Video Database

This supplementary material has been provided by the authors to give readers additional information about their work.

© 2022 Kugener G et al. *JAMA Network Open.*

eMethods. Supplemental Methods

Cadaveric Simulation Details

Resident, fellow, and attending surgeons, including neurosurgery, and otorhinolaryngology–head and neck, were recruited for participation at nationwide educational courses between 2017 and 2020 (the North American Skull Base Society Annual Meeting, North American Skull Base Society Summer Skull Base Surgery Course, University of Southern California Annual Hands-On Comprehensive Neuro-Endoscopy Course, Emory Cranial Base Surgery Course, and Stryker Med-Ed Skull Cranial Surgery Course). The study was approved by the IRB of the University of Southern California.

A high-fidelity simulated operating room was constructed, including a surgical technician, surgical field, and simulated patient vital signs. A lightly embalmed human cadaveric head was prepared, and a standard endonasal endoscopic approach to the sella turcica was performed by study staff. Following cadaver perfusion at a standardized flow rate and physiological blood pressure using an artificial blood substitute, injury of the cavernous segment of the internal carotid artery (ICA) was induced by laceration. Participants were given standardized verbal instructions on the parameters, instruments, and goals of the simulation, but were not initially given specific technical instructions.

The protocol consisted of trial 1 (T1), followed by an educational intervention, and then trial 2 (T2) was performed. During T1 and T2, participants attempted to control the perfused ICAI using a variety of standard instruments and techniques (suction, cottonoid patties, and, ultimately, muscle patch control). Monitors showed simulated vital sign decompensation, and each trial ended when either hemostatic control was obtained using a muscle patch or simulated mortality occurred at 300 seconds (defined as ‘trial failure’). Time to hemostasis (TTH, in seconds) and blood loss (BL, in mL) were evaluated for each trial. After T1, subjects received specific feedback from one of the

© 2022 Kugener G et al. *JAMA Network Open.*

course instructors (endoscopic endonasal approach experts) and watched a standardized video of a senior author (G.Z.) explaining the recommended stepwise technique of ICAI management. T2 was then performed with feedback.

Data Set Development

Intraoperative video was taken from the Karl Storz Video Neuro-Endoscope used during each of these trials. A total of 147 videos from this nationwide educational intervention were recorded and saved. Videos were recorded at a frame rate of 30 frames per second (fps) and a resolution of 1280x720 or 1920x1080. These videos are taken from multiple cadaveric heads, with different lighting, anatomy, laceration sites, camera resolutions, and brands of endoscopic instruments.

The duration of the trials varies from 46 seconds to 5 minutes. Each trial video was downsampled from 30 frames per second (fps) to 1 fps using ffmpeg and a bounding box was created around each surgical instrument in each frame and labeled as suction, grasper, cottonoid, muscle, string, drill, scalpel, or other (non-specified surgical instruments). For each instance of an instrument in frame, an annotator drew a bounding box around the instrument such that the entirety of the instrument was encompassed by the bounding box, following published protocols using the open-source image annotation software VoTT. Following a first pass of video annotations, members of the research team with significant experience viewing endoscopic endonasal video (GK, DJP) manually audited annotations to check for quality. Frames with missing annotations or mislabeled annotations were subsequently re-annotated

In conjunction with trial video recordings, “outcomes data” (e.g. blood loss, task success) and demographic data (e.g. training status, confidence) was recorded for each participant.

Model Development

Using published model weights from pretraining on ImageNet, RetinaNet and YOLOv3 were fine-tuned to perform instrument bounding box detection on the SOCAL data set. When designing our training, validation, and test splits, we used one cohort of surgeons as a test set (7 surgeons, 14 trials), another cohort as a validation set (6 surgeons, 9 trials), and the remaining cohorts for model training (5 cohorts, 63 surgeons, 135 trials). We chose to split the data set in this way to replicate a real-world workflow, where the model would be tasked to analyze video (split into individual frames) from an entirely new cohort of surgeons. We evaluated these models on their ability to assign bounding box coordinates to all instances of suction, grasper, cottonoid, string, muscle, drill, scalpel, and other instruments in our data set.

We inputted our data set into two publicly available object detection models, RetinaNet and YOLOv3.[37,38] We trained and evaluated these models on their ability to assign bounding box coordinates to all instances of suction, grasper, cottonoid, string, muscle, drill, scalpel, and other instruments in our data set. The input into these object detection models is a video file, and the output is the bounding box coordinates and label of any of the eight instruments we trained on. To implement RetinaNet, we forked the fizyr/keras-retinanet github repository, initialized the model, using the preexisting Imagenet weights, and trained the model for 45,000 iterations (18 epochs, 2500 steps, batch size of 1). To implement YOLOv3 we forked the zzh8829/yolov3-tf2 github repository using the preexisting Darknet weights and trained the model for 26,972 iterations (11 epochs, 2,452 steps, batch size of 8). The learning rate was initialized to 1e-5 for RetinaNet and 1e-3 for YOLOv3. For both models, the learning rate was decreased by a factor of 10 whenever the loss plateaued for two epochs. Training was stopped when the loss plateaued for five consecutive epochs.

© 2022 Kugener G et al. *JAMA Network Open.*

eTable 1. Publicly Available Tool-Annotated Surgical Video Data Sets Across Surgical Specialties

	No. Frames (No. unique videos/trials)	Surgical Domain	Intraoperative vs Benchtop	Annotation styles	No. Instruments	Outcomes
SOCAL[33]	31,443 (147)	Endoscopic endonasal	Intraoperative, cadaveric training exercises	Instrument bounding boxes	8	Yes (success/failure, time to hemostasis, blood loss, surgeon experience demographics
EndoVis 2017[12]	1,800 (8)	Robotic Surgery (Abdominal)	Intraoperative video of nephrectomy (porcine model)	Instrument segmentation	8	No
Cholec80[10]	86,000 (80)	Laparoscopic surgery (Abdominal)	Cholecystectomy (real intraoperative video)	Phases (all 80 videos)<br>Bounding Boxes (10 videos, tool tip)	7	No
ATLAS Dione[13]	22,467 (99)	Robotic Surgery	Benchtop Model	Bounding Boxes (tool tip)	2	Yes (Surgeon experience status)
JIGSAW[14]	Unspecified (103)	Training exercise	Benchtop model	Instruments position and kinematics	Gestures: 15<br>Instruments: 2 (left and right)	Yes (surgical skill (GRS (modified OSATS), surgeon prior experience)
Neurosurgery[15]	2,476 (14)	Microscopic Surgery (Neurosurgery)	Intraoperative video (Brain tumor/spine tumor removal)	Instrument Segmentation	8	No
CADIS[11]	4671 (50)	Microscopic Surgery (Ophthalmology)	Intraoperative video of cataract surgery	Instrument segmentation	Instruments: 3*<br>Anatomy: 4	No
SimSurgSkill (EndoVis 2021)[20]	Unspecified	Robotic Surgery	VR Simulation (RAS)	Instrument bounding box	Instruments: 2	Yes (3, Objective skill metrics)
SARAS-ESAD/MESAD[19]	33,398 (4)	MIS, Complete radical prostatectomy (RARP)	Intraoperative	Action bounding boxes	21 Actions	No
ROBUST-MIS (EndoVis 2019)[21,22]	10,040 (30)	Proctocolectomy, rectal resection, sigmoid resection	Intraoperative, minimally invasive surgical procedures	Instrument segmentation, phases	17	No

*Data set described instrument (1), surgical tape and retractors separately; they are combined in the table for clarity.

© 2022 Kugener G et al. *JAMA Network Open.*

eTable 2. Baseline Demographic and Outcome Characteristics of Surgeon-Trials Contained in the SOCAL Video Database

	Overall	Attendings	Trainees
Surgeons	75	25	48
Neurosurgery	38	7	31
Otorhinolaryngology	35	18	17
Avg Years of Surgical Experience (SD)	7 (5.5)	12 (6.2)	4 (1.8)
Avg Endoscopic Cases 12 months (SD)	15 (19.8)	31 (24.5)	7 (9.9)
Avg Cadaveric Cases 12 months (SD)	3 (4.7)	4 (3.9)	3 (5)
Carotid Confidence Pre-Exercise (SD)	2.4 (1.2)	3 (1.2)	2 (1.1)
Carotid Confidence Post-Exercise (SD)	3.5 (1.1)	4 (1)	3.3 (1.1)
General Confidence Pre-Exercise (SD)	3.5 (1.2)	4.4 (0.8)	3.1 (1.1)
General Confidence Post-Exercise (SD)	3.7 (1.2)	4.6 (0.6)	3.3 (1.2)
Real ICAI? No. (%)	20 (27)	12 (48)	8 (17)
Simulated ICAI? No. (%)	16 (22)	9 (38)	7 (15)
Real or Simulation ICAI? No. (%)	24 (33)	13 (54)	11 (23)
Avg. Trial 1 TTH (SD)	239.5 (84.5)	205.4 (90.7)	257.3 (76.1)
Avg. Trial 2 TTH (SD)	157.9 (85.5)	139.7 (79.6)	168 (87.9)
Difference (95% CI) [% Improvement]	79 (52-106) [34]	66 (16-115) [32]	86 (54-119) [35]
Avg. Trial 1 BL (SD)	531.6 (374.4)	342.8 (315.3)	630 (367.5)
Avg. Trial 2 BL (SD)	377.3 (388.6)	257.7 (295)	443.8 (420.3)
Difference (95% CI) [% Improvement]	154 (65-243) [29]	85 (-48-218) [25]	192 (73-312) [30]
Trial 1 Success No. (%)	33 (45)	17 (68)	16 (33)
Trial 2 Success No. (%)	61 (84)	23 (92)	38 (79)

ICAI = internal carotid artery injury. TTH = time to hemostasis (seconds). BL = blood loss (mL).
